# Implementing a Real-time Workplace-based Assessment Data Collection System Across an Entire Medical School’s Clinical Learning Environment

**DOI:** 10.15694/mep.2021.000022.1

**Published:** 2021-01-25

**Authors:** Reem Hasan, Carrie Phillipi, Andrea Smeraglio, Jessica Blank, Alexandra Shuford, Cameron Budd, Amy Garcia, Patricia Carney

**Affiliations:** 1 Oregon Health & Science University

**Keywords:** workplace-based assessment, competency based medical education, entrustable professional activities

## Abstract

This article was migrated. The article was marked as recommended.

**Background & Objectives:** Workplace-based assessments (WBAs) are a vital aspect of medical student competency assessment for the core Entrustable Professional Activities (EPAs), but pose significant challenges since assessment must occur in real-time during the routine care of patients. We developed an online WBA system designed to overcome these challenges, and implemented it across an entire undergraduate medical education program to address the need for EPA competency assessment. We describe the development and implementation process, and present initial results from our inaugural medical student cohort.

**Methods:** The WBA tool was designed to be student-driven, easy to use, and minimally disruptive to clinical care. Students trigger assessments by choosing the desired EPA to be assessed within a custom-built Qualtrics ^XM^ survey application. Their clinical assessor is prompted to select their level of involvement in the activity using the modified Ottawa co-activity scale and provide brief written feedback. Direct verbal feedback at time of discussion is encouraged.

**Results:** 3,568 WBAs were completed. The mean number of assessments per student for all EPAs combined was 24.27 with a range of 1-103. All students completed at least one WBA. Over the course of 12 months, the mean number of EPAs recorded per student in this cohort was lowest for EPA 10 (Recognizing a Patient Requiring Urgent or Emergent Care and Initiate Evaluation and Management) (mean=0.36; range 0-4; n=53) and was highest for EPA 6 (Provide an Oral Presentation for a Clinical Encounter) (mean=5.46; range 1-17; n=803). The mean number of minutes it took to complete the assessments was 2.7 minutes with a standard deviation of 1.2 minutes (n=2,803).

**Conclusion:** An electronic application-based survey collecting real-time WBAs to assess progress toward attaining competence in EPA performance resulted in increased assessment data within a medical school cohort.

## Introduction

Undergraduate medical education (UME) has traditionally relied on a time-based model with multiple summative assessments resulting in a letter grade after the completion of a time requirement ( Holmboe *et al.*, 2010). The movement to competency-based medical education (CBME) represents a significant shift towards a more holistic focus on learner progression based on readiness rather than time. This provides more complete support for the spectrum of learners, including early identification of struggling learners so that individualized academic plans can be developed, to early graduation of learners progressing at a rapid pace ( Ross *et al.*, 2018; Carraccio *et al.*, 2016). CBME necessitates that educators commit to extensive assessments. One framework for CBME in UME is the AAMCs 13 core Entrustable Professional Activities (EPAs), a series of physician tasks which students are expected to be proficient at by graduation.

Workplace-based assessments (WBAs), obtained during the routine care of patients ( Swanwick and Chana, 2009; Miller, 1990) is one way to accomplish CBME evaluation. This approach allows for both context-based assessment and real-time feedback allowing learners to reflect upon and make immediate change to their practice ( Sabey and Harris, 2012; Hicks *et al*., 2018; Hast Prins, Gjedde Brønd, Malling, 2019; Norcini and Burch, 2007). Challenges to WBA include a lack of programmatic and workplace integration of assessment, and hesitancy of students to interrupt patient flow and request faculty time to perform assessments ( Saedon *et al*., 2012). Most studies of WBA have been conducted during residency training ( Hicks *et al*., 2018; Kogan *et al*., 2017) or outside the United States ( Hast Prins, Gjedde Brønd, Malling, 2019; Wilkinson *et al*., 2008; Mathew *et al*., 2018; Barrett *et al*., 2016). Furthermore, the majority of prior WBA efforts focus on a single discipline rather than programmatic wide implementation.

In our efforts to move toward CBME, we implemented a health system-wide WBA online data collection application across the entire undergraduate medical education program. We utilized the EPA framework with the goal of providing additional assessments for graduation. The purpose of this paper is to describe the experience of WBA development and implementation in an academic health center.

## Methods

### Educational Setting

Oregon Health & Science University (OHSU) is a 522 bed teaching hospital, biomedical research facility and level 1 trauma center that serves as the safety net hospital and academic health center for the state of Oregon and southwest Washington. The *YourMD* competency-based integrated medical school curriculum at OHSU launched in 2014 and replaced the previous traditional two years pre-clinical and two years clinical-discipline based curriculum. Woven throughout the curriculum are eighteen “threads” ranging from ethics, informatics, communications, and safety to professionalism and clinical reasoning. The curriculum is divided into two phases: Foundations of Medicine (FOM) and the Clinical Experience Phase (CEP).

Throughout the FOM curricular phase, students undergo multiple low and high stakes assessments consistent with CBME. Assessment data are available to students, their coaches, and the assessment team using a secure password-protected portfolio system with a viewing portal. Once students complete the first phase of the curriculum and pass Step 1, they advance to the CEP. Similar to the FOM, multiple points of assessment are undertaken, including midterm formative comments, knowledge-based quizzes, history and physical evaluations, presentations, self-reflections, and procedure logs, all of which are included in the portfolio system. Direct, real-time assessment data were not systematically collected before WBA implementation.

### Implementation Team

WBAs were based on the 13 AAMC EPAs, and two groups were tasked with overseeing EPA development and implementation: the Core EPA Pilot Committee and the Entrustment Group. Members of the Core EPA Pilot Committee were responsible for representing OHSU in the AAMC national EPA pilot and developing recommendations from their concept groups, which included leaders in faculty development, curriculum and assessment, and entrustment as well as student and resident liaisons. The Entrustment Group was charged with developing processes and procedures to review student data to make decisions about EPA competency attainment. It was comprised of UME and graduate medical education (GME) leaders selected by application. Key UME staff were integrated into both groups. All faculty members of the Core EPA Pilot and Entrustment Committee were tasked with faculty development efforts and received funding (0.1 to 0.15 FTE) to support their work.

### Development and Use of the WBA Online Tool

The WBA tool was designed as a student-driven system that would be easy to use and minimally interrupt clinical care. This resulted in a smart phone or tablet-based application (app) that could be handed to an evaluator for completion ( Appendix 1). The app was built using Qualtrics ^XM^ survey software (Seattle, WA). The Qualtrics app had the added benefit of being functional while offline, so WBAs could be collected with or without access to internet, and available on all user platforms. We were further able to program custom functions (such as a signature line) into the survey. This software was also licensed to OHSU for IT and Qualtrics support.

Students initiate assessments using the WBA app on their phone or tablet and are prompted to choose the desired EPA for assessment, followed by the clinical discipline, and the evaluation setting (e.g., inpatient). Students then select assessor type (faculty member, resident, fellow, other). The students hand their device to the clinical assessor who is prompted to select their involvement in the activity was using a modified version of the Ottawa Co-Activity Scale ( Gofton *et al*., 2012) **.** Finally, the evaluator is prompted to type or dictate one or two specific comments about how the student could improve, then the WBA is digitally signed, submitted, and uploaded to the server. Once submitted, students cannot change the feedback or score; a signature line and confirmatory email help verify legitimacy.

Submitted WBAs can be viewed via the same secure password-protected portfolio described earlier. The Research and Evaluation Data for Educational Improvement (REDEI) system was developed by a team of educational researchers and leaders in conjunction with a software programmer at OHSU. REDEI is a relational database that uses Structured Query Language (SQL) to display data. Data from multiple assessment systems, including Qualtrics ^XM^, is exported into REDEI using Application Programming Interfaces (API). Students, coaches, advisors, and administrative leaders can see learner performance metrics relative to the cohort mean for all assessments using the personalized REDEI viewing portal. The REDEI system and all data collection processes have been reviewed and approved by OHSU’s Institutional Review Board (IRB#10873). In addition, a Certificate of Confidentiality protects data in the REDEI System from forced disclosure.

### Curriculum Integration

WBA requirements were implemented step-wise into the medical school curriculum to increase acceptability and to ease the transition for current students and faculty. As new students matriculate, EPAs and WBAs are emphasized as larger components of their pre-clinical and clinical education. The number of WBAs required to graduate increases depending on the year of matriculation. For the class described in this report, they were required to complete 50 WBAs before graduation.

To further ensure students gain competency in all EPAs, students are required to complete at least two WBAs for each EPA, while encouraged to complete as many WBAs in as many of the EPAs as possible to demonstrate competency. Each core clerkship also adopted two EPAs of focus. Students target WBA completion to specific clerkship-emphasized EPAs. WBAs can be completed by any supervisor and in any clinical environment, including both clinical cores and electives. Requirements starting with the class of 2022 include that at least 50% of the WBAs must come from attendings as opposed to residents, fellows, or other clinical staff.

Pre-clinical students are first introduced to WBAs in a course titled “Introduction to Preceptorship.” Students receive a two-hour, large group lecture facilitated by faculty members of the EPA committee. EPAs and WBAs are introduced conceptually, students practice using the Qualtrics app, troubleshoot technical challenges, and simulate WBAs with fellow students using pre-defined scenarios ( Appendix 2). WBAs are reintroduced prior to the start of clinical rotations in a large group, two-hour long lecture that is similar in scope and content to the lecture provided in the pre-clinical years.

### Faculty Development

Faculty development efforts followed recommendations published from the Core EPA Pilot ( Favreau *et al*., 2017), and focused on creating a shared mental model for entrustment for UME educators. Didactic presentations and interactive workshops were held at University and Departmental levels detailing the Core EPAs and principles of trust. Within these presentations, WBAs and directions about how to use the smart phone application were described. To extend our message, we utilized a “train the trainer” approach. Core clinical experience directors (clerkship directors), curriculum committee leaders, and coaches were trained and encouraged to underscore the importance of WBAs at departmental faculty meetings and meetings with trainees. These efforts were tracked to ensure all departments were reached.

Core EPA and Entrustment Committee members also produced educational materials, which were distributed electronically throughout the University including our off-site practice locations. These included a narrated presentation, didactic presentations, workshops and a one-page tip sheet. The workshops and videos allowed for deliberate practice of WBAs and encouraged discussions about entrustment. Core EPA Pilot Members provided presentations at every GME orientation to capture the residents and fellows.

We also relied on students to assist with faculty development, recognizing it would be difficult to reach every clinical supervisor. Student workshops were held at orientation, and prior to preceptorship and formal clinical experiences. Included were practical tips to improve reciprocal feedback, and pocket cards describing how to solicit and receive feedback developed by students at Vanderbilt University, another Core EPA Pilot site, were distributed.

### Data Analyses

A total of 3,599 WBAs were submitted by 148 students of the 2021 graduating class during the first 12 months of their clinical experiences. Of these, 31 (<1%) did not include either an EPA or level of assessment and were excluded, leaving 3,568 assessments for inclusion in analyses. Though this cohort officially includes 155 students, upon which the demographic characteristics are based, seven students (4.5%) had left the cohort to work on masters or other doctorate degrees as part of their program. Descriptive statistics, including frequencies, percentiles, means, standard deviations, and ranges (minimum, maximum counts), were calculated.

## Results/Analysis

The average age of medical students included in this study was 29 (24-41 years). The majority were female (54.8%), white (69.7%), not of Hispanic origin (92.9%), single (81.9%) and were not parents (96.8%) ( Table 1).

**Table 1.** Demographic Characteristics of Medical Student Cohort (n=155)

Characteristics	Value	%
**Mean Age in Years (SD †)** *Range*	29 (3.07) 24-41	
**Gender** Male Female	**n** 70 85	**%** 45.2 54.8
**Race** American Indian/Alaska Native Asian/Pacific Islander Black White Mixed Race	**n** 1 28 3 108 15	**%** 0.61 8.1 1.9 69.7 9.7
**Ethnicity** Hispanic Origin Non-Hispanic Origin	**n** 11 144	**%** 7.1 92.9
**Marital Status** Single Married/Partnered Divorced	**n** 127 26 2	**%** 81.9 16.8 1.3
**Parental Status** Yes No	**n** 5 150	**%** 3.2 96.8

† SD=Standard Deviation

3,568 WBAs were completed. The mean number of EPAs recorded per student ( Table 2) was lowest for EPA 10 (Recognizing a Patient Requiring Urgent or Emergent Care and Initiate Evaluation and Management) (mean=0.36; range 0-4; n=53) and was highest for EPA 6 (Provide an Oral Presentation for a Clinical Encounter) (mean=5.46; range 1-17; n=803).

**Table 2.** Mean Number Student Assessments per EPA (n=148 students; 3,568 Assessments)

	Mean Number of Assessments per Student (n=148)	
EPA	Mean (SD)	Range
Gather a Hx and Perform PE (n=525)	3.57 (2.59)	0-13
Prioritize a Differential Dx Following a Clinical Encounter (n=288)	1.96 (1.74)	0-11
Recommend and Interpret Common Diagnostic and Screening Tests (n=161)	1.10 (1.22)	0-7
Enter and Discuss Orders and Prescriptions (n=240)	1.63 (1.96)	0-14
Document a Clinical Encounter in the Patient Record (n=462)	3.14 (2.64)	0-14
Provide an Oral Presentation of a Clinical Encounter (n=803)	5.46 (3.60)	1-17
Form Clinical Questions and Retrieve Evidence to Advance Patient Care (n=204)	1.39 (1.38)	0-6
Give or Receive a Patient Handover to Transition Care Responsibility (n=110)	0.75 (0.98)	0-5
Collaborate as a Member of an Interprofessional Team (n=346)	2.35 (1.88)	0-9
Recognize a Patient Requiring Urgent or Emergent Care and Initiate Evaluation and Management (n=53)	0.36 (0.77)	0-4
Obtain Informed Consent for Tests and/or Procedures (n=58)	0.39 (0.79)	0-4
Perform General Procedures of a Physician (n=231)	1.57 (2.25)	0-19
Identify System Failure and Contribute to a Culture of Safety and Improvement (n=87)	0.59 (0.90)	0-5
Mean number of observations per student for ANY EPA (All EPAs combined (n=3,568)	24.27 (14.28)	1-103

**Figure 1.**
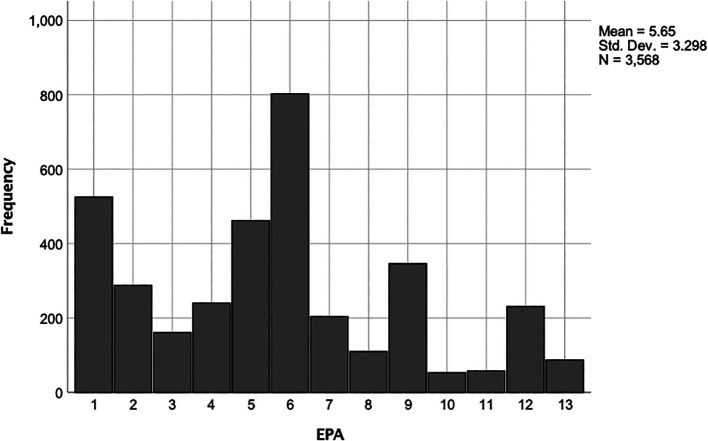
Frequency of Assessments According to EPA

The mean number of total EPA assessments per student was 24.27 (range 1-103). 100% of students in this cohort completed at least one WBA. In addition, the mean number of minutes it took to complete the assessments was 2.7 minutes with a standard deviation of 1.2 minutes (n=2,803) ( *data not shown*). Figure 1 shows the distribution of the EPAs according to frequency.

**Figure 2.**
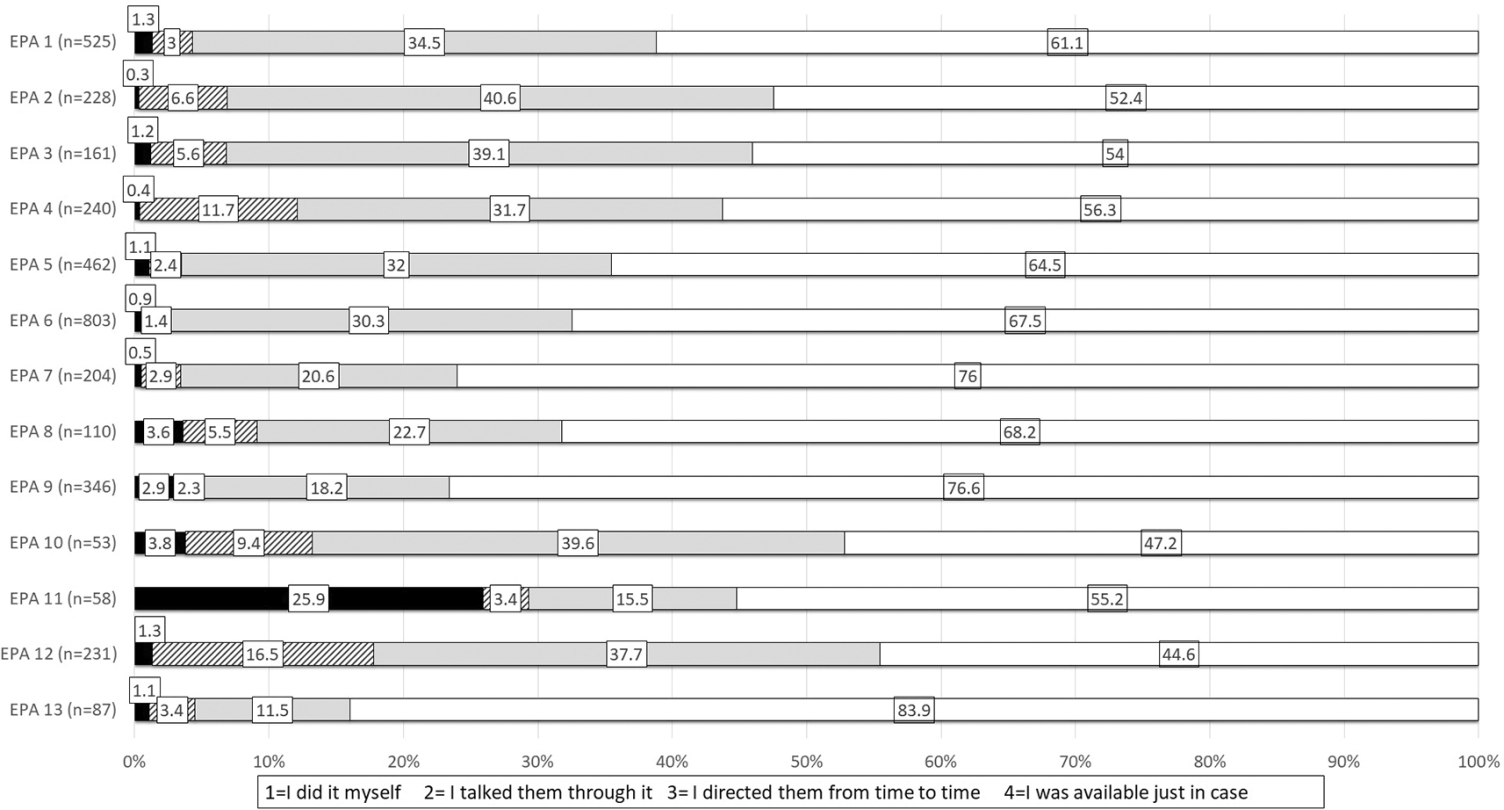
Level of Supervisor Involvement According to EPA

Figure 1, 2, and Table 4 EPA Legend:

1. Gather a Hx and Perform PE
2. Prioritize a Differential Dx Following a Clinical Encounter
3. Recommend and Interpret Common Diagnostic and Screening Tests
4. Enter and Discuss Orders and Prescriptions
5. Document a Clinical Encounter in the Patient Record
6. Provide an Oral Presentation of a Clinical Encounter
7. Form Clinical Questions and Retrieve Evidence to Advance Patient Care
8. Give or Receive a Patient Handover to Transition Care Responsibility
9. Collaborate as a Member of an Interprofessional Team
10. Recognize a Patient Requiring Urgent or Emergent Care and Initiate Evaluation and Management
11. Obtain Informed Consent for Tests and/or Procedures
12. Perform General Procedures of a Physician
13. Identify System Failure and Contribute to a Culture of Safety and Improvement

EPA 6 (Provide an Oral Presentation of a Clinical Encounter) was most frequently assessed and EPA 10 (Recognize a Patient Requiring Urgent or Emergent Care and Initiate Evaluation and Management) was least frequently assessed. Figure 2 illustrates the level of supervisor involvement according to EPA.

Supervision Level 4 (I was available just in case) was most commonly recorded for all EPAs with a range of 44.6% for EPA 12 (Perform General Procedures of a Physician) to 83.9% for EPA 13 (Identify System Failure and Contribute to a Culture of Safety and Improvement). Level 3 supervision (I directed them from time to time) was second most common with a range of 11.5% for EPA 13 (Identify System Failure and Contribute to a Culture of Safety and Improvement) to 40.6% for EPA 2 (Prioritize a Differential Dx Following a Clinical Encounter). Level 2 supervision (I talked them through it) ranged from 1.4% for EPA 6 (Provide an Oral Presentation of a Clinical Encounter) to 16.5% for EPA 12 (Perform General Procedures of a Physician), and Level 1 (I did it myself) ranged from 0.3% for EPA 2 (Prioritize a Differential Diagnosis Following a Clinical Encounter) to 25.9% for EPA 11 (Obtain Informed Consent for Tests and/or Procedures). Table 3 shows students’ WBAs according to clinical discipline and setting.

**Table 3.** EPA Student Assessments According to All Clinical Disciplines and Clinical Setting

	Clinical Setting							
Clinical Discipline	ED	ICU	Inpatient ward	L & D	OR/Procedural	Other	Outpatient clinic	*Total*
Anesthesiology	0 (0.0%)	0 (0.0%)	1 (6.7%)	0 (0.0%)	13 (86.7%)	0 (0.0%)	1 (6.7%)	15
Colon & Rectal Surgery	0 (0.0%)	2 (8.0%)	20 (80%)	0 (0.0%)	2 (8.0%)	0 (0.0%)	1 (4.0%)	25
Dermatology	0 (0.0%)	0 (0.0%)	0 (0.0%)	0 (0.0%)	0 (0.0%)	0 (0.0%)	2 (100%)	2
Emergency Medicine	7 (70.0%)	1 (10.0%)	0 (0.0%)	0 (0.0%)	0 (0.0%)	0 (0.0%)	2 (20.0%)	10
Family Medicine	7 (1.2%)	1 0.2%)	12 (2.0%)	2 (0.3%)	1 (0.2%)	1 (0.2%)	583 (96.0%)	607
Internal Medicine	4 (0.6%)	4 (0.6%)	626 (96.0%)	0 (0.0%)	0 (0.0%)	6 (0.9%)	12 (1.8%)	652
Internal Medicine Subspecialty	0 (0.0%)	5 (15.6%)	12 (37.5%)	0 (0.0%)	0 (0.0%)	8 (25.0%)	7 (21.9%)	32
Neurological Surgery	1 (4.5%)	3 (13.6%)	13 (59.1%)	0 (0.0%)	1 (4.5%)	0 (0.0%)	4 (18.2%)	22
Neurology	2 (0.4%)	95 (19.2%)	338 (68.4%)	0 (0.0%)	0 (0.0%)	9 (1.8%)	50 (10.1%)	494
Obstetrics & Gynecology	5 (1.4%)	0 (0.0%)	69 (19.5%)	123 (34.7%)	50 (14.1%)	1 (0.3%)	106 (29.9%)	354
Orthopedic Surgery	0 (0.0%)	0 (0.0%)	0 (0.0%)	0 (0.0%)	1 (16.7%)	0 (0.0%)	5 (83.3%)	6
Otolaryngology (Head and Neck Surgery)	0 (0.0%)	0 (0.0%)	0 (0.0%)	0 (0.0%)	7 (87.5%)	0 (0.0%)	1 (12.5%)	8
Pathology	0 (0.0%)	0 (0.0%)	0 (0.0%)	0 (0.0%)	0 (0.0%)	1 (50.0%)	1 (50.0%)	2
Pediatrics	0 (0.0%)	54 (12.1%)	251 (56.2%)	0 (0.0%)	0 (0.0%)	14 (3.1%)	128 (28.6%)	447
Pediatric Subspecialty	0 (0.0%)	15 (13.5%)	71 (64.0%)	0 (0.0%)	0 (0.0%)	4 (3.6%)	21 (18.9%)	111
Psychiatry	2 (0.5%)	2 (0.5%)	411 (94.3%)	0 (0.0%)	1 (0.2%)	7 (1.6%)	13 (3.0%)	436
Radiology	0 (0.0%)	0 (0.0%)	0 (0.0%)	0 (0.0%)	0 (0.0%)	0 (0.0%)	1 (100%)	1
Surgery	33 (9.9%)	5 (1.5%)	147 (44.1%)	0 (0.0%)	95 (28.5%)	1 (0.3%)	52 (15.6%)	333
Surgical Subspecialty	1 (9.1%)	1 (9.1%)	5 (45.5%)	0 (0.0%)	3 (27.3%)	0 (0.0%)	1 (9.1%)	11
*Total*	62 (1.7%)	188 (5.3%)	1, 976 (55.4%)	125 (3.5%)	174 (4.9%)	52 (1.5%)	991 (27.8%)	3568

Assessments were submitted by 19 distinct clinical disciplines. Family medicine assessments predominated in the outpatient clinic (n=583 or 58.8% of all assessments completed in the outpatient setting). Internal medicine assessments were common in inpatient setting (n=626 or 31.7% of all assessments done in inpatient settings). Neurology (n=95) and pediatrics (n=54) predominated in ICU, while surgery (n=33) was more common in the emergency department. Table 4 shows the EPAs according to core clinical disciplines with the grey shading indicating EPAs planned to be a focus of that clerkship.

**Table 4.** EPAs According to Core Clinical Discipline

Core Discipline	EPA1	EPA2	EPA3	EPA4	EPA5	EPA6	EPA7	EPA8	EPA9	EPA10	EPA11	EPA12	EPA13	*Totals*
Family Medicine†	104 (17.1%)	69 (11.4%)	31 (5.1%)	56 (9.2%)	98 (16.1%)	92 (15.2%)	21 (3.5%)	10 (1.6%)	30 (4.9%)	14 (2.3%)	8 (1.3%)	62 (10.2%)	12 (2.0%)	607
Internal Medicine †	66 (10.1%)	76 (11.7%)	53 (8.1%)	45 (6.9%)	51 (7.8%)	151 (23.2%)	50 (7.7%)	15 (2.3%)	53 (8.1%)	16 (2.5%)	17 (2.6%)	24 (3.7%)	35 (5.4%)	652
Neurology †	87 (17.6%)	26 (5.3%)	20 (4.0%)	50 (10.1%)	40 (8.1%)	148 (30.0%)	23 (4.7%)	10 (2.0%)	64 (13.0%)	4 (0.8%)	1 (0.2%)	12 (2.4%)	9 (1.8%)	494
Obstetrics & Gynecology †	51 (14.4%)	10 (2.8%)	7 (2.0%)	9 (2.5%)	62 (17.5%)	93 (26.3%)	17 (4.8%)	28 (7.9%)	28 (7.9%)	2 (0.6%)	5 (1.4%)	40 (11.3%)	2 (0.6%)	354
Pediatrics †	61 (13.6%)	53 (11.9%)	20 (4.5%)	26 (5.8%)	47 (10.5%)	152 (34.0%)	28 (6.3%)	10 (2.2%)	38 (8.5%)	0 (0%)	3 (0.7%)	6 (1.3%)	3 (0.7%)	447
Psychiatry †	74 (17.0%)	28 (6.4%)	12 (2.8%)	31 (7.1%)	99 (22.7%)	40 (9.2%)	31 (7.1%)	20 (4.6%)	64 (14.7%)	4 (0.9%)	5 (1.1%)	16 (3.7%)	12 (2.8%)	436
Surgery †	52 (15.6%)	13 (3.9%)	9 (2.7%)	9 (2.7%)	39 (11.7%)	75 (22.5%)	18 (5.4%)	4 (1.2%)	33 (9.9%)	7 (2.1%)	10 (3.0%)	55 (16.5%)	9 (2.7%)	333
Other *	30 (5.7%)	13 (4.5%)	9 (5.6%)	14 (5.8%)	26 (5.6%)	52 (6.5%)	16 (7.8%)	13 (11.8%)	36 (10.4%)	6 (11.3%)	9 (15.5%)	16 (6.9%)	5 (5.7%)	245
*Totals*	525	288	161	240	462	803	204	110	346	53	58	231	87	3568

* Column %

† Row %

**Figure 3.**
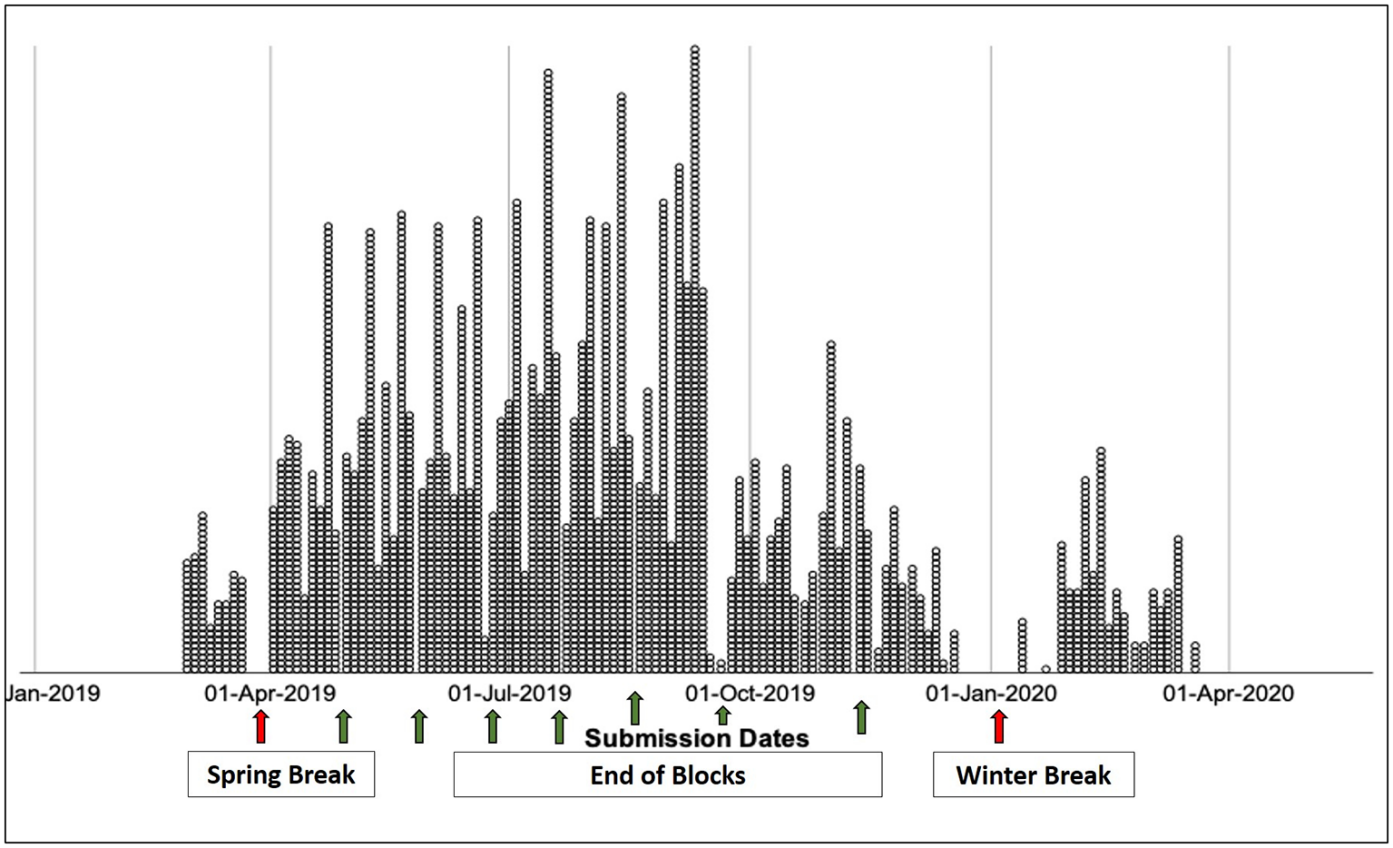
Dot Plot of EPA Assessment Submission Date

Planned assessments ranged from 1.1% for psychiatry and EPA 11 (Obtain informed consent for tests and procedures) to 26.3% for OB/GYN and EPA 6 (Provide oral presentation of a clinical encounter). WBAs were obtained nearly exclusively in the core clinical experiences (93.5%). **Figure 3** shows EPA submission dates, starting in March of 2019, peaking in October of 2019, and ending in March of 2020 when medical students were removed from clinical sites due to the COVID-19 pandemic.

## Discussion

In this report, we describe the implementation of WBA for all 13 AAMC EPAs across an institution. Utilizing an online smartphone app, we developed, integrated, and disseminated use of WBAs according to an EPA framework across a wide variety disciplines and settings. With sufficient faculty and student development, WBAs can be collected at scale. This adds to what has been previously demonstrated on a smaller scale in individual clerkships ( Fazio *et al*., 2018).

Our students were able to complete a large variety of EPAs during their clinical experience. Not surprisingly, learners more often solicited gathering a history and physical exam, documentation in the electronic medical record, and giving an oral presentation as these skills are highly emphasized in clinical training. The least commonly assessed WBAs focused on handoff, informed consent, quality/safety, and initiating urgent/emergent care treatment. These are less likely to be emphasized or tracked by assessors in core rotations in our curriculum and the lack of WBA attainment in these areas may indicate curricular and/or assessment gaps. EPA 10 (initiating urgent/emergent care treatment), was the only EPA not tied a core clerkship requirement, potentially lowering its assessment frequency. Furthermore, this cohort of students had not completed their ICU/ED rotations where this task would be more commonly encountered.

We noted fewer reports of WBAs scored at level 1, 2 compared to level 3, 4. We believe this occurs because students are more likely to request an assessment when they feel well prepared. However, we plan to further investigate this trend as it could also represent other entities, such as characteristics of the assessor.

Nineteen different clinical disciplines assessed students through this inaugural WBA implementation, demonstrating breadth of uptake in varied settings, potentially aided by faculty and student development. This was likely further supported by linking specific EPAs, and therefore WBAs, to specific clerkships. This focused students and assessors on specific skills during a given rotation and may have increased uptake of some of the less common EPAs. This will need to be elucidated in future research.

After an initial period of student and faculty education, we noted that the rate of WBA completion was relatively stable week-by-week over the subsequent 6 months. In the early fall, the requirements for WBAs changed and we saw a decrease in completion rates. This suggests student-initiated assessments are associated with medical school expectations.

We did note several issues related to the technical implementation of this tool, which warrant further discussion. To accommodate supervisors who did not want to use a mobile phone to complete the evaluation, access to the survey was also provided via web link. Students voiced concerns about privacy on their personal mobile phones, such as if notifications appeared when a supervisor was filling out an evaluation. To address this, we recommended they use either ‘Airplane’ or ‘Do not disturb’ mode to prevent notifications from appearing. We also considered liability issues if a personal phone was dropped or broken while used for this purpose, and the SOM developed a fund to replace phones broken during a WBA and not covered by insurance.

Strengths of this study include our ability to successfully implement a WBA app across clinical training in an entire medical school and the resultant large numbers of WBAs for analysis. However, this study reflects the experience of a single institution and may not be generalizable to other institutions. In addition, results of a single year from a single cohort of students were included, and so lacks benefits of more years of data collection as students progress. There may be bias in the timing of student collection of WBAs and choice of assessors leading to a “good job” grade rather than a true reflection of competency. We have very little data for several EPAs (particularly 10, 11 and 13). The WBAs may showcase existing parts of our curriculum and should be considered within context of the entire spectrum of competency-based education. Further research is still needed to more fully characterize the utility and timing of WBAs in medical education.

## Conclusion

Implementation of WBA according to the EPA framework is possible within a cohort of students in a medical school-based health system. We plan to report on the contributions of WBA to decisions around entrustability in EPA performance, academic advancement, and graduation.

## Take Home Messages

• We describe the experience of WBA development and implementation in an academic health center
• Implementation of WBA according to the EPA framework is possible and results in meaningful assessment within a cohort of students in a medical school-based health system

## Notes On Contributors

**Reem Hasan**, MD PhD, is Assistant Professor in the Departments of Internal Medicine and Pediatrics at Oregon Health & Science University. Her medical education research interests include early longitudinal student experiences, student engagement, and active, experiential learning. Clinically, she is interested in clinic transformation and high functioning primary care medical homes. ORCID ID: https://orcid.org/0000-0002-8557-0656

**Carrie Phillipi** MD, PhD is a Professor of Pediatrics and the Vice Chair of Education in the Department of Pediatrics at Oregon Health & Science University. Her research interests include competency-based medical education and newborn medicine. Related to these interests, she has participated in the Association of American Medical Colleges Core Entrustable Activities for Entering Residency Pilot and is the Co-Managing Director of the Better Outcomes through Research for Newborns Research Network. ORCID ID: https://orcid.org/0000-0002-3921-1847

**Andrea Smeraglio** MD is an Assistant Professor in the Department of Internal Medicine at OHSU. Andrea Smeraglio, MD is an Assistant Professor in the Department of Internal Medicine at Oregon Health & Science University and in the Division of Hospital and Specialty Medicine at the Portland Veterans Affairs Medical Center where she works clinically as a hospitalist. Her educational and research pursuits focus around the intersection of Improvement Science and Medical Education. Specifically, how we prepare the next generation of trainees to create and practice safe, quality and high-value healthcare. ORCID ID: https://orcid.org/0000-0003-0775-7930

**Jessica Blank** is a fourth year medical student at Oregon Health & Science University. She will pursue a residency in Internal Medicine during the 2021 residency application cycle. Her medical education research interests include student evaluation, incorporating advocacy in the curriculum, and developing leadership skills. ORCID ID: https://orcid.org/0000-0001-8500-3697

**Alexandra Shuford**, PhD, is the Director of Undergraduate Medical Education Assessments in the School of Medicine at Oregon Health & Science University. Her medical education research interests include assessment and evaluation methods, epistemology of evidence based medicine, and active learning methods. Her background includes online pedagogy, faculty development, and teaching undergraduate and graduate students in liberal arts. ORCID ID: https://orcid.org/0000-0002-2839-3016

**Cameron Budd**, BS, is a data analyst and systems administrator in the Oregon Health & Science University School of Medicine. His involvement in medical education includes system administrator for MedHub, Qualtrics, and Ilios. ORCID ID: https://orcid.org/0000-0002-8833-6566

**Amy Garcia** MD is Assistant Dean for Student Affairs at Oregon Health & Science University School of Medicine, Undergraduate Medical Education and Associate Professor in the Department of Pediatrics, Division of Pediatric Gastroenterology. Her medical education interest is in coaching, advising, and mentorship along with diversity, equity, and inclusion. Clinically, she is interested in small bowel rehabilitation, nutrition, and cystic fibrosis. No ORCID available.

**Patricia Carney,** PhD, MS is Professor of Family Medicine at Oregon Health & Science University’s School of Medicine. She has doctoral training in educational psychology and her educational research interests span undergraduate and graduate medical education to continuing professional development of physicians post training. She has more than 280 publications in these areas. ORCID ID: https://orcid.org/0000-0002-2937-655X.

## Appendices

### Appendix 2. Scenarios Used During Large Group WBA Introductions

• Internal Medicine - You are on the second week of your Internal Medicine Core Clinical Experience. A new intern just started on the service that day. She is getting to know her 10 patients. You ask her to provide a WBA for you. Her pager is going off non-stop and a nurse just came into the team room to tell her she incorrectly entered a medication order for one of her patients. What should you do in this situation?
• Pediatrics - You are on your third week of your Pediatrics Core Clinical Experience. You solicit a WBA from your hospitalist (ward) attending who agrees to do it, and you tee up the survey and hand your phone to your attending. Right then, a code blue is called and the attending gives you back your phone and rushes to help the patient in distress. What should you do in this situation?
• Neurology - You are on your first week of your Neurology Core Clinical Experience. Earlier that week, you successfully solicited 2 WBAs from your attendings in clinic. Today, you solicit a WBA from your senior resident on the consult team who has not heard about this before. He looks at you with puzzled look. He is not mean, he just has never heard of it before. What should you do in this situation?
• Family Medicine - You are working in an outpatient clinic on your fourth week of your Family Medicine Core Clinical Experience. You want to solicit a WBA from your preceptor today, but you see that she is far behind schedule due to a previous patient needing to be admitted to hospital earlier in the afternoon. She has previously been open and receptive to completing many of the WBAs for you, but you sense she is stressed and her last two patients have each commented that they have been waiting a long time to see her today. What should you do in this situation?
• Obstetrics/Gynecology - You are on your OB/Gyn Core Clinical Experience. Today you are working with a partner of your main preceptor in the Gynecology Clinic. This partner loves to teach students and has been showing you all sorts of interesting clinical findings and having you take the lead on interviewing the patients while he observes you in clinic. You think about soliciting a WBA from him but all of your other WBAs during this clinic time have been with your main preceptor. What should you do in this situation?
• Psychiatry - You are on your Psychiatry Core Clinical Experience and you just worked with your senior resident on how to enter orders into EPIC for a complicated patient. Your resident had you enter and then PEND, for her to log in and sign them. You want to solicit a WBA for EPA 4 but it is not one of those linked to core, and you have not gotten your 3 WBAs yet this week. What should you do in this situation?
• Surgery - You are on your first rotation, the Surgery Core Clinical Experience. You go a whole week and no one ever asks you if they can provide a WBA to give you feedback. What’s up with that? What do you do in this situation?
• Multi-disciplinary Rural Rotation - You are on a rural rotation in an outpatient clinic in Ruraltown, Oregon where your Family Medicine physician preceptor has asked that you spend a day with the clinic Social Worker to better understand the work that the other members of the interprofessional team do. The Social Worker and you work all day together where he helps you understand the roles and responsibilities he has in caring for patients in the clinic, as well as how he interacts with other members of the care team. Toward the end of the day, you take the lead in the conversation with the Nursing and Medical Assistant staff members regarding a care plan for a patient who has met with you and the Social Worker. Even though EPA 9 (Collaborate as a Member of an IPE Team) is not a linked EPAs in this course, can you solicit a WBA from the Social Worker?
