# Supplementary material for: Implementing a Real-time Workplace-based Assessment Data Collection System Across an Entire Medical School’s Clinical Learning Environment
Source: MedEdPublish (2016). 2021 Jan 25;10:22. [Version 1] doi: 10.15694/mep.2021.000022.1 (PMC10939594; doi:10.15694/mep.2021.000022.1)
Supplement: Peer review history

# Reviewer report: Reviewer response for version 1

**DOI:** 10.21956/mep.18843.r26854

**Published:** 2021-03-20

**Authors:** Megan Anakin

**Affiliations:** 1 University of Otago

This review has been migrated. The reviewer awarded 4 stars out of 5 Thank you for publishing this study about how your team implemented and evaluated a workplace-based assessment tool at your institution. Due to the ongoing challenges of providing students with adequate learning and assessment opportunities during the COVID19 pandemic, I found your study very helpful to consider how my institution might better provide assessment feedback to our students while creating useful assessment records for supporting progress decisions. I appreciated the many sources of data that were considered in your study. To enhance the significance and implications of your tool development and use to readers, please consider revisiting some of the literature presented in the introduction again in the discussion section. Please also consider making further connections to the literature to emphasise the usefulness of this tool to address well-documented concerns such as providing students with quality feedback and gathering authentic assessment data. Please consider discussing how educators in other national contexts might be able to develop a similar tool for their institutions.I would be very happy to read and review a revised version of this article.

Reviewer Expertise:

NA

No decision status is available

# Reviewer report: Reviewer response for version 1

**DOI:** 10.21956/mep.18843.r26855

**Published:** 2021-01-25

**Authors:** Balakrishnan Nair

**Affiliations:** 1 Centre for Medical Professional Development and University of Newcastle

This review has been migrated. The reviewer awarded 4 stars out of 5 This is an excellent paper describing the implementation of WBA in a medical school. WBA is all about shifting the assessment to the top of the Miller’s pyramid; to do this, it needs paradigm shift, both from the learners and assessors. So well done to the teamFaculty and students were trained regarding this philosophy assessment. The tenet of WBA about multiple tools and multiple assessors was met, and the electronic data collection worked out well tooEven though the original intention was to do 50 WBAs, the mean number achieved was around 24, still better than the traditional assessment ( I am not sure whether I got it right, reading the article ). The mean assessment duration was 2.7 minutes which was considerably shorter than the standard time taken for WBA assessments like mini CEX. The strength of the WBA is immediate constructive feedback and I am not sure how many minutes were taken to do this .Students may select “soft “ assessors, even though this may even out, when done by multiple assessors Overall the team should be proud of their achievements to introduce the WBA to the curriculum

Reviewer Expertise:

NA

No decision status is available
